# The Importance of Social Connectedness in Building Age-Friendly Communities

**DOI:** 10.1155/2012/173247

**Published:** 2011-11-24

**Authors:** Charles A. Emlet, Joane T. Moceri

**Affiliations:** ^1^Social Work Program, University of Washington Tacoma, Tacoma, WA 98401, USA; ^2^Nursing Program, University of Washington Tacoma, Tacoma, WA 98401, USA

## Abstract

The purpose of this paper is to further elucidate the importance of social relationships and social connectedness with aging in place and in developing elder-friendly communities. The process used in this study was inclusive of younger adults (age 40–65) as well as older adults (65+) in order to further understand how they envision a community that could support their own aging in place. A community forum, using the World Café format, was conducted in order to engage community members, 40 years and older, in conversation about the importance of social connectedness in elder-friendly communities. A second purpose of this forum was to obtain data on what would keep aging boomers in their community as they age. Three major themes emerged from qualitative analysis of the forum: *social reciprocity*, *meaningful interactions*, and *structural needs/barriers*. The results of this study reinforce the importance of social connectedness in creating and maintaining elder-friendly communities for older adults, as well as soon-to-be retired individuals, wishing to maintain life connectedness to their community. The study suggests the possibility of using more nontraditional research techniques (such as the World Café process) for gathering community level data.

## 1. Introduction

Increasingly, gerontological researchers, practitioners, policy makers, and planners are concerning themselves with the growing importance of aging in place. Aging in place does not have one single definition but broadly is considered to be the ability to continue to live in the environment of one's choice, even when declining competence reduces or threatens independence [1], while allowing for consumer choice in the types of services delivered [2]. Lawler [3] suggests that aging in place strategies can minimize inappropriate care and work best as a comprehensive and holistic approach to the needs of aging individuals and communities. Lau and colleagues [4] have conceptualized a framework for aging in place safely and acknowledge the importance of multiple factors, including the biological and psychological characteristics of the individual, the network of social support, formal services, the need for medical services, and the structure of the home and neighborhood. This and other frameworks clearly recognize that aging in place strategies must consider not only the personal (micro) environment, including housing, but also community and structural components as well [4, 5].

## 2. Theoretical Frameworks

Before embarking on a discussion of elder-friendly communities, it is important to discuss a number of theoretical frameworks and conceptualizations from gerontology that help inform our understanding of aging in place. There are numerous frameworks that are relevant to aging in place including ecological theory, person in environment, and social inclusion/exclusion. In addition, the area of environmental gerontology has specific relevance to this discussion. 

 Ecological theory [6] suggests that there is a mutual relationship and mutual reciprocity between individuals and their environment and that this interaction occurs at multiple levels, including the micro-, exo-, mezzo-, macro-, and chronosystems levels [6]. Ecological theory is important for the concept of aging in place as it suggests that individuals interact with multiple “levels” of environment in their day-to-day lives. Older people must not only interact with microenvironments such as their home and immediate family, but also with broader systems that can equally influence their ability to age in place. Another theoretical perspective that informs our discussion is that of person in environment [1]. This perspective, like ecological theory, acknowledges that the environment interacts with individuals at multiple levels and suggests that the environment is not a static backdrop but rather continually changes. From the person-in-environment perspective, the older person must continually take from the environment what he or she needs, control what can be modified, and adapt to conditions that cannot be changed [1].

 Also of relevance to this discussion is the theory of social inclusion/exclusion. In social gerontology, the theory of social inclusion/exclusion examines the role of older people and highlights the social costs when individuals, families, or communities are excluded from or become disengaged from larger society due to characteristics such as poverty, gender, ethnicity, or neighborhood [7]. Scharf and colleagues [8] conceptualize the inclusion and exclusion of older people as associated with three key themes: participation and integration (beyond the labor market), spatial segregation, and institutional disengagement. Of particular interest in our exploration of aging in place is the thematic area of participation and integration. Scharf et al. [8] posit that participation and integration not only include older people's involvement in community life, but also are associated with their social capital, including civic participation, and the nature of social networks and mutuality/reciprocity. An elder-friendly community can support these concepts.

In addition to several theoretical frameworks, the field of environmental gerontology has specific relevance to the topic of aging in place. Wahl and Weisman [9] suggest that environmental gerontology's (EG) theories and findings can and should influence the development of age-friendly communities. For example, EG is concerned with the role of neighborhoods and the influence those neighborhoods have on opportunities and constraints of residents [10]. At a more macrolevel, EG recognizes the community as a locus of aging with a sociophysical and policy perspective [11]. 

With regard to elder-friendly communities, we can draw upon the work of Lawton [12] who posited that the environment has three major functions of maintenance, stimulation, and support [9]. Maintenance is concerned with the consistency and predictability of one's environment, while stimulation is concerned with the effect of stimuli on behavior. Finally, support is concerned with the environment's potential to compensate for diminished or lost competencies [13]. 

## 3. Elder-Friendly Communities

In recent years, the concept of elder-friendly communities has become central to the notion of aging in place. Described in various ways, an elder-friendly community is a place where “people can live their entire lives, if they so desire, rather than having to relocate and lose their social capital” [14, page 6]. An elder-friendly community examines the environment in more macro-level terms as places where older people are actively involved, valued, and supported by an infrastructure that accommodates their needs [15]. In what was perhaps the first on-line conference focusing on elder-friendly communities, the Sierra Health Foundation suggested that elder-friendly communities are those communities in which age is not considered a barrier to improving lifelong interests and activities, where support and accommodations exist to meet the basic health and social needs of those with age-related disabilities, and where opportunities exist for older adults to develop new sources of fulfillment and engagement [16]. 

 While the literature on elder-friendly communities is to a degree embryonic, several models have been developed in recent years. Among these models created in the United States, Canada, and Europe, the interrelatedness of social and structural factors is found to be consistently important. For example, Feldman and Oberlink's [17] work on the *AdvantAge Initiative* demonstrated that elder-friendly communities must address basic needs, optimize well-being, maximize independence, and promote civic engagement. The City of Calgary Elder-Friendly Community Project noted that feeling safe, being valued and respected, staying active, and building community were important elements of an elder-friendly community [18]. The World Health Organization (WHO) has established international guidelines for age-friendly communities that include the encouragement of active aging by optimizing opportunities for health, participation, and security in order to enhance people's quality of life as they age [19]. According to the WHO, an age-friendly city adapts its structures and services to be accessible to, and inclusive of, older people with varying needs and capacities. 

While various models have emerged identifying aspects key to the concept of elder-friendliness, a consistent theme found in the literature is associated with social interaction or social connectedness. Scharlach [14] suggests that an elder-friendly community fosters both *connection *and *contribution*. An elder-friendly community will assist older adults to maintain social connectedness while deepening existing relationships. Such a community will recognize the social capital of these relationships that in turn result in *contribution.* The concept of contribution recognizes the wisdom and experience of older citizens and sees them as more than clients, but rather as active contributors to community well-being [14]. Similarly, The Calgary Project identified as important the active participation of older people in their communities. This premise is consistent with the work of Rubinstein and colleagues [20] who found that the ability to actively manage one's environment was a source of well-being for older adults. Similarly, the model of age-friendly communities developed by the WHO clearly recognizes that social participation and social support are strongly associated with overall well-being, allowing elders to exercise their competence and enjoy the respect and esteem of their community [19]. Alley and colleagues [15] remind us that a community's respect for older adults, which includes available opportunities, contributes significantly to their quality of life. 

While social participation and connectedness are important in an elder-friendly community, there is a need for reciprocity between older adults and their community. For example, the *AdvantAge Initiative* [17] promotes the importance of civic engagement, including meaningful connections, volunteer and paid opportunities, and the prioritization of aging issues. The WHO acknowledges that an age-friendly community provides the option for older adults to continue to contribute to their community through civic engagement with both paid and volunteer opportunities and to have the ability to be active in the political process. The benefits of such reciprocity are many, such as an increased sense of purpose and satisfaction for older adults as they engage with the community, while younger community members may benefit from the knowledge and experience older adults bring to the community. As an example, intergenerational programs recognize the knowledge and skills possessed by older adults that can be shared with youth, while providing opportunities for civic engagement for the older person [1]. The key here is mutual benefit while recognizing that each segment benefits differentially. 

 Much of the research on elder-friendly communities has highlighted the multidimensional nature of community life and has not focused primary attention on social connectedness despite the importance of interdependence and engagement as primary qualities of aging in community [21]. For example, the *AdvantAge Initiative* identifies social and civic engagement but used quantitative measures to evaluate communities in three preordained realms [17]. Additionally, age-friendly community projects often obtain views from current elders. If an age-friendly community is a positive place to “grow” old, then the views of younger citizens (baby boomers, for example) need to be taken into account. Alley and colleagues [15] suggest that in an age-prepared community, processes of planning and advocacy are utilized to foster aging in place, which may be a prospective view of what is needed in planning for future community needs. This process must take into account the views and needs of the citizens who are not yet defined as older adults, but who will bring their own needs and views to the community. 

The purpose of this paper is to further elucidate the importance of social relationships and social connectedness in developing an elder-friendly community. The process used in the project described here was inclusive of younger adults (age 40–65) as well as older adults (65+) in order to help understand how they envision a community that could support their own aging. Alley et al. [15] describe the importance of an “age-prepared” community [15, page 8] as one which has assessed its current services and is planning for the needs of future populations. Second, the qualitative methodology used in this study allowed for a more naturalistic and personal narrative. Padgett [22] acknowledges the importance of “meaning making” in the narrative process that includes storytelling, conversation, and discourse of naturally occurring speech. This study, therefore, was informed by the perspective of narrative analysis and the use of the spoken and written word in narrating the meaning of social connectedness as we age. 

## 4. Background

 In April of 2002, surveys related to assessing the elder friendliness of communities were completed by 5.100 individuals, 65 and over, throughout 10 cities across the United States [23]. In one participating community in Western Washington, a total of 514 surveys were completed. Findings suggested that older adults in that community were satisfied with their neighborhoods and participated in religious or cultural activities, and the majority of respondents were engaged in health screening [24]. The vast majority of these respondents had participated in some type of social activity in the past week and slightly fewer than one in three people volunteered [23]. The survey results were promising and positive, yet are now dated and do not reflect the opinions of members of the aging baby boom generation. Second, the original survey did not focus specifically on the issue of social connectedness but limited the focus to volunteering and participation in cultural and religious activities. 

Recognizing the need to better refine and focus attention on the importance of social connectedness as part of elder-friendly communities, a city committee responsible for the continuation of the elder-friendly community agenda sponsored a community forum in October of 2009. A community forum using the World Café format [25] was conducted in order to engage community members, 40 years and older, in conversation about the importance of social connectedness in elder-friendly communities. Previous research in this area has approached the topic of social connectedness through an a priori definition of social engagement, primarily utilizing quantitative methods for measurement and evaluation [23]. This forum, however, sought to understand social connectedness from those approaching retirement using a more naturalistic method. A second purpose of this forum was to obtain data on what would keep aging boomers in their community as they age. The results of the forum and its applicability to elder-friendly communities and aging in place research are being presented here.

### 4.1. World Café As a Research Strategy

The World Café is a concept that was born out of *Appreciative Inquiry* [26], which is a form of research that emphasizes the positive aspects of an experience, particularly how that experience can foster creativity among people [26]. The World Café format involves exchanging ideas and sharing different points of view in a safe, intimate setting with the purpose of coalescing wisdom and experience into learning. A foundational component of the World Café concept is conversations, purposeful conversations that have a reason for taking place, “conversations that matter” [25, page 4]. They may be initiated to solve a community problem or to envision a preferred future, in this case an elder-friendly community, with a focus on social connectedness. The World Café format places an emphasis on moving from simply talking to taking action. This movement takes place as participants are able to understand the connection between talking and acting, or conversation as action [25]. It was in this context of “sharing collective discoveries” [25, page 138] that the community forum took place. This study provided an opportunity to test the value of the World Café format as a method for future research.

## 5. Methods

This study was determined to be an exempt study by the University of Washington Human Subjects Division. The method employed for this study involved a melding of the World Café format as the structure of the study with a focus group format as the process that informed data collection in the study. Qualitative methodology was then used for data analysis. The data collection procedures differed from traditional focus groups in some significant ways. First, groups formed, discussed, and reformed with different participants for each of the three main questions that were posed at the forum. Second, instead of the more customary audio or video taping of the groups, each table was covered with paper on which participants wrote and/or drew as they discussed the topic at hand. These notes and doodles became the transcript along with notes taken by each table leader. This is consistent with narrative analysis in which both spoken and written words are used in meaning making [22]. Finally, groups were given great latitude as to how they addressed the discussion topic for their table. Some groups created action plans, and others were more reflective. The discussion leaders at each table helped to keep the group on topic and were careful not to inject their opinions into the group discussion. 

The setting for the study was a community forum for those over 40 years of age living within the school district boundaries of a suburban community in Western Washington with a population of approximately 37.000, whose residents are predominately Caucasian (87%). Approximately 32% are ages 45 and over [27]. The forum included refreshments, and people were invited to sit at one of several round tables covered with paper for writing thoughts as they occurred to the participants. The conversation at each table began with the posing of one of three questions, with ample time allowed for each table group to discuss, strategize, and imagine a preferred future in an elder-friendly community. The three questions were as follows (1) What does it mean to you to be socially connected? (2) How can our city help with life transitions that would keep you in this community? (3) What do I have to offer my community? These three questions were developed through consensus by the city level committee charged with examining issues and processes that enhance an age-friendly community. The questions were designed to determine how people define and make meaning of being socially connected, to identify aspects of community life that would reinforce continuity with the community versus relocation to another community after retirement, and to ask participants to think about their own value to the community, thereby initiating thought around the idea of social reciprocity. Conversation was not limited to only the question at hand, and participants were invited to speak, draw, and write about the broader topic throughout the session. At set times, participants were asked to move to a different table, to be with a different group of people, and to consider a different question, until all three main questions were answered by most of the participants. One member from each table stayed behind during the rotation in order to serve as an ambassador for the previous members, thus assisting in continuity of conversation. A goal was to allow participants to engage creatively as they tackled the questions together. So, rather than gather individual feedback, table leaders encouraged participants to converse with each other and to spend time thinking together about potential solutions to dilemmas as they were raised by group members. Once the group session was completed, participants were invited to gather into a large group to debrief and discuss the most important topics from the perspectives of the participants. This conversation was also guided, and notes were recorded.

### 5.1. Sample

A purposive sample of people over age 40 was recruited through newspaper ads and invitations from the city Parks and Recreation Department and through the Aging in Place Committee (AIP) membership. Membership lists from the Senior Activity Center and local faith communities also served as sources for potential participants. The invitation requested community members to participate in a community forum to discuss how to create, promote, and maintain a more elder-friendly community. Ultimately, 23 individuals participated in the community forum and ranged in age from midforties to late eighties. Participants therefore represented both those who might be identified as baby boomers as well as those who are currently retired and may be defined more traditionally as older adults. We did not collect specific data on age, but some participants offered their age as part of the conversations. The majority of participants were female and Caucasian. Since this was originally conceived by the AIP committee as a community forum and not a research project, no additional sociodemographic data were collected on socioeconomic status, education, or other typical variables associated with creating a demographic profile.

### 5.2. Data Analysis

Following the World Café community forum, researchers were asked to analyze the data from the event in order for the AIP committee to present findings and make recommendations to city government officials. No identifying information about participants was included with the data provided for analysis. Using an approach consistent with grounded theory [28], the researchers analyzed the data for common categories and themes. First, they met together and carefully reviewed the data from each of the questions. They used an open coding process for notes of verbal exchanges, drawings and notes from participants, and memos from group leaders. The few illegible writings and unrecognizable doodles were dismissed from the analysis process. As categories began to emerge, coding became more selective until three main themes were identified. Throughout analysis, the researchers engaged in conversation about meanings and interpretations, until they were satisfied they had a clear understanding of the data. In order to confirm that trustworthiness of the data was maintained, once the themes were identified, the AIP committee reviewed the findings and then invited all of the original forum participants to attend a focus group to discuss the findings. The focus group was held in the same location as the community forum approximately two months after the forum was convened and was made up of five individuals (approximately 20% of forum members). Like the forum participants, most focus group participants were female and Caucasian, with one or two individuals representing communities of color. Focus group participants also ranged in age from early 50s to mid-70s. The focus group participants reviewed, clarified, and added data to the transcripts and confirmed that the themes identified by the researchers were reflective of the community meeting. The review by the focus groups provided credibility and trustworthiness (validity) to the qualitative findings, reinforcing a fit between the respondents' views and the researchers' interpretation as well as being confirmatory, for example, demonstrating that the study's findings were not imagined [22]. This process, known as member checking, not only serves to validate findings but is empowering to the participants and reinforces the close relationship between the researchers and the informants in qualitative research [22].

## 6. Results

 The researchers identified three major themes that emanated directly from the data and were confirmed by the focus group. All three themes emerged from the open coding and were ultimately labeled as follows: *social reciprocity*, *meaningful interactions*, and *structural needs/barriers*. The three themes were identified and confirmed by both boomers and older participants.

### 6.1. Social Reciprocity

This theme was directly related to the overarching focus on social connectedness but illustrated the importance of added value in these relationships. Within the theme of social reciprocity, giving and receiving to/from one's community were both seen to be of equal importance. Some participants were currently volunteering or communicated an interest in doing so (giving). While exact ages were not available, it appeared that older adults (65+) were more likely to be active volunteers than their younger counterparts. Baby boomers expressed interest in volunteerism, while older adults may have already engaged in that process if they were interested. Many participants expressed an interest in receiving through such things as enhanced educational opportunities (e.g., more age-friendly options from the local community college and public university). The idea of educational opportunities at no or low cost was initially mentioned by younger participants. Participants also indicated that venues for creating social connectedness could come from both *formal *and *informal* entities. Formal entities are those which would require some infrastructure involving an organization or business, such as theater, outdoor concerts, or free movie nights. An example might be the initiation of social activities through city government, the local Chamber of Commerce, or even a local business. Informal entities would include activities that require limited resources, such as the creation of book clubs or neighborhood gatherings. Participants also suggested that such activities aimed at increasing social connectedness could be *sponsored *or *influenced* by community resources. For example, through the Senior Activity Center, the city might sponsor a new boomer or senior walking group. Communities also could advocate for the development of social venues through influence. The city government, for example, could attempt to influence the policy of a not- for- profit community organization regarding how cumbersome and degrading the process is for older adults with limited income to obtain reduced membership fees. 

Reciprocity between formal and informal systems could also occur. For example, a nongovernmental organization such as a church could recruit older volunteers from their congregation to volunteer in local schools. The theme of social reciprocity can and should conceptually occur at multiple levels, such as between governmental and nongovernmental organizations, as well as between individuals and their community. In all aspects of the data, reciprocity (the mutual exchange of commercial or other privileges) was exemplified as the willingness to give and receive in order to foster social connectedness. No one suggested getting something for nothing. Inherent in the discussion of social reciprocity was the notion that the relationship between the individual older persons may occur at multiple levels of community and environment. Relationships and mutual exchange might occur at the level of neighborhood, a community organization, or at the level of city government or policy advocacy. For example, some forum participants suggested helping others by providing space for a communal garden (neighborhood), while some suggested that developing a volunteer position to work as a senior ombudsman related to negotiating city services would be beneficial to the whole (city government level). This exchange improves the well-being of those being helped while fostering a sense of accomplishment and service. 

### 6.2. Meaningful Interactions

While participants discussed the desire to give and receive in order to maintain social connectedness, they were clear, however, that these experiences should be meaningful both to themselves and others. While a high number of forum participants expressed a desire to volunteer in their community, they clearly stated that the activity should be meaningful to them and important to the community. This sentiment communicates the view that these individuals see themselves as having social capital (whether or not it is recognized by others). Volunteerism was seen as an important way to give back to the community. As one participant put it, “we should all volunteer, even if it is in the home—respite, visitor, chores.” The participants shared a collective view that the purpose of volunteering was not to kill time. Rather, participants were interested in sharing their passions, time, sense of history, and even sharing personal space to accomplish this end. One participant suggested that people share their gardens with others or help others to do crafts in their homes. Participants also viewed volunteering as a way they could advocate for others and for their community. Finally, if participants were to be involved in meaningful interactions through volunteering, they wanted to feel appreciated for the work they did. They voiced the concern that organizations often diminished or ignored the value of their time as volunteers and took volunteers for granted. It is important to note here that forum participants did not suggest they wanted to volunteer for the sake of recognition, but rather they felt the need to be valued—not taken for granted. The message that was communicated by forum participants was that they desired both the organization/community in which they served as well as themselves to view their contributions as meaningful. While speculative due to a lack of specific data on age, the older participants appeared more settled in their roles as volunteers, as many of them had held these roles for some time. Younger adults (boomers) appeared to have more concerns about the meaning they derived from volunteer opportunities and how that may be accomplished. 

### 6.3. Structural Needs/Barriers

While the majority of participants provided feedback on what or how they could contribute to their community to enhance social connectedness, a similar number of people voiced substantial frustration with the lack of either organized opportunities or communication with potential organizations with which to volunteer. These issues were impediments to social reciprocity as well as to meaningful interaction, and as such were labeled as structural needs or barriers. Structural (infrastructural) needs or barriers were those things participants viewed as currently lacking in the community but, if present, would facilitate social reciprocity both in terms of physical and social venues. For example, many forum participants expressed the need for improved methods by which potential volunteers could be connected to opportunities (community entities). These sentiments were expressed more strongly by younger participants. The examples that were given included organizations that needed volunteers should return phone calls more promptly to potential volunteers, as well as the need for more personal connections between those requesting volunteers and the people who might be willing to give of their time. Again, the importance of the value of time was communicated by the participants. They were not interested in having to make numerous inquiries to potential organizations in order to volunteer. The feeling expressed was that there was a lack of reciprocity from the very beginning on the part of agencies or organizations with which these individuals might wish to volunteer. 

Transportation was described as an additional structural barrier and was mentioned frequently in all table conversations. Transportation was viewed as an essential element of social connectedness. In areas of both volunteerism as well as overall social connectedness, transportation issues associated with public transit and walkable communities were voiced. Issues concerning transportation included that a lack of reliable, frequent, and accessible transportation created barriers for participants within the community. As one individual said, “[a] lack of transportation isolates seniors.” The view communicated by these participants was that improved transportation can foster and enhance social connectedness by decreasing barriers of distance and reducing the need for use of one's personal vehicle. One important distinction between younger and older participants was noted relevant to transportation. While younger participants voiced interest in improved transportation as a means toward improved social connectedness and as an environmentally friendly alternative to automobiles, older participants expressed a more urgent need for improved transportation, as well as having a more specific personal need. For example, one couple who was likely in their 70s expressed the need for improved transportation services for their parents (in their 90s) as they identified gaps in transportation services as personally problematic. 

## 7. Discussion

 The purpose of this research project was to analyze data gathered from aging individuals (including baby boomers) on the importance of social connectedness in the creation of elder-friendly communities through a naturalistic method of inquiry. By engaging in a more naturalistic conversation utilizing the World Café format, the participants in the study were able to utilize conversation in meaning making without the confines of any a priori assumptions about social connectedness. 

 The findings from this community forum and the subsequent focus group reinforce earlier data from the original *AdvantAge Initiative* as well as other literature on elder-friendly communities and point to the utility of several important theories. First, these findings echo the original framework from the *AdvantAge Initiative* [17], which emphasizes the importance of social and civic engagement. The individuals from this community forum, as well as the elder counterparts in the original study, underscored the importance of meaningful connections to family, friends, and neighbors as part of civic engagement. An elder-friendly community needs to find new ways to promote active and continual engagement in community life. The findings from this study parallel the view of Scharlach [14] who suggests that as “we get older and ever closer to the end of our lives, maintaining social connectedness and deepening existing relationships becomes a priority” [14, page 9]. These findings also reinforce the importance of participation and integration, which is a critical element of social exclusion theory [8]. Forum participants identified multiple activities associated with social inclusion/exclusion including production (economic or socially valued) activity, political activity to improve or protect the social environment, and social activity that involved engagement with family, friends, and community. Scharf and colleagues [8] define participation and integration as “older people's embeddedness in social networks and the extent to which older people contribute to or draw upon social capital that exists in their neighborhoods” [8, page 316]. Thus, our findings related to social reciprocity appear consistent with the major theme from social inclusion/exclusion theory. Our findings also reinforce the importance of Lawton's [12] environmental function of support. A community needs to be dynamic in order to support changes in the older citizenry. While the concept of support is typically relevant to adjustment to altered or lost competencies, the concept of support can be extended to include the need for continued and changing modes of social and civic engagement. 

 This study also reinforces both the importance of volunteer opportunities and that those opportunities be purposeful and meaningful. As suggested by Scharlach [14], in an elder-friendly community, older adults are not just seen as clients or passive recipients of services, but “active contributors to the well-being of the community” [14, page 9]. In the original *AdvantAge* survey, residents from this community volunteered at a rate substantially lower than the national average for the 10 *AdvantAge* communities [23]. What we learned from this study was that aging community members held interest and motivation to volunteer or otherwise be engaged in their community. We believe they see themselves as having social capital [29], but as Putnam [29] points out, others may not always share their view. The environmental function of stimulation [12] is relevant here as participants seemed to look to their community for stimuli for enhanced social well-being and to elicit new and relevant social and leisure behaviors [9]. Older participants appeared more likely to have volunteer and community activities in place, while younger adults (boomers) were perhaps seeking out methods for accomplishing that goal. Both younger and older participants also noted structural barriers to social connectedness and social integration, supporting Alley et al. [15] who suggest that while communities may be able to support aging in place, they may also contain barriers that make community living difficult for older residents. A recent study of 253 older adults reinforces the importance of organizational structure in volunteerism. Tang et al. [30] found organizational support (defined as choice of volunteer activity, training, and ongoing support) to be associated with socioemotional benefits, including perceived contribution and personal benefits. These researchers concluded that the “psychological well-being of older adults can be improved through engagement in meaningful volunteer activities and contribution to others” [30, page 603], again reinforcing what Rubinstein and colleagues [20] noted concerning the connection between well-being and active environmental management. In order for these benefits to occur, however, an elder-friendly community must work to eliminate structural and organizational barriers to volunteerism and social connectedness. As Scharf and colleagues [8] assert, participation and integration are enhanced by good public service such as access to reliable transportation. To not provide such services serves to reinforce the social exclusion of older people. The identification of structural barriers also reinforces the person-in-environment perspective that the needs of older people change over time and must be successfully navigated in order to maintain social integration. Lawton's [12] environmental function of maintenance is relevant here. If a community is to be elder-friendly, the infrastructure needs to be consistent and predictable at the very least, while at the same time dynamic in its ability to provide stimulation and support. 

 Community-based research is particularly useful when it is able to identify problems and move toward a resolution of that issue. Researchers can partner with communities to study areas of interest, interpret results [31], and assist in the empowerment of community members to make changes [32]. The findings from this study have already resulted in community level change efforts related to volunteerism. An annual volunteer fair was initiated in 2010 with the goal of creating a venue to match older volunteers with community level volunteer opportunities. This newly formed activity grew directly out of the identification of structural barriers in this research and was created through a partnership of senior advocates, the community's AIP committee, and local organizations, including the area hospital. In the first year of operation, 30 community organizations and programs participated along with 120 attendees. More than 80% of older adults were successfully matched with local organizations, thus improving social connectedness, integration, and reciprocity in a direct and clear way. This event has now been established as an annual event sponsored by seniors, city government, and other community entities. Its goal is to improve civic engagement among older residents, thus fostering the connectedness between older residents and organizations that serve the community.

 In addition to the importance of civic engagement, the philosophy of aging in place supports the continued importance of maximizing independence for not only the frail and disabled, but for aging adults of all abilities. In particular, these findings point to the need for accessible and available transportation, an issue that city officials and community advocates should attempt to improve through partnerships. As Feldman and Oberlink [17] noted in their original findings, “transportation and safety are fundamental factors that enable older adults to stay connected to the community” [17, page 5]. Rosenbloom [33] suggests that transportation in elder-friendly communities will need to be planned to provide more customized services, linking residential concentrations with important destinations, including volunteer opportunity destinations. The project findings noted that while all participants voiced the need for improved transportation services, the kinds of services desired may change with age. The lack of this kind of transportation was clearly identified as a major structural barrier reinforcing social exclusion and needs to be considered as future planning takes place. With impending cuts to public transportation, the aging in place committee is examining potential alternatives to improve transportation through private and voluntary means. 

### 7.1. Limitations

 The results of this study provide important information on social connectedness in elder-friendly communities. Still, this study has several limitations that must be acknowledged. First, as a qualitative and naturalistic study, the findings are the specific views of those individuals involved and cannot be generalized to any larger population of aging adults. Second, a further limitation is that those who responded to the invitation to participate in the community forum may have had a greater interest in the topic, or a vested interest in having their voices heard as compared to those who did not or could not attend. However, the study results provide a new dimension to the subject area and support previous studies and theories on aging in place, thus adding to the picture of what needs to be done to support the creation of elder-friendly communities. Because of the homogeneous makeup of forum participants, the voices of other communities such as communities of color were not clearly heard. It must be acknowledged that the opinions and concerns of this group do not likely represent all older adults in this community. Finally, because sociodemographic data was not collected on individuals, distinctions between older (65+) and younger participants are based upon educated guesses about participant's age.

## 8. Conclusion and Implications

 The results of this study reinforce the importance of social connectedness, participation, and integration in creating and maintaining elder-friendly communities and suggest that the findings are areas of concern not just for the old-old, but for recent and soon-to-be retired individuals wishing to maintain life satisfaction. The study suggests the possibility of using more nontraditional research techniques for gathering community level data such as the kinds of findings generated from the World Café process. While creating and fostering elder-friendly communities can be a long and ongoing process, small incremental change can occur from such studies as is illustrated by the case of the annual volunteer fair now established in this community. 

 If a national agenda of enabling our aging population to age in place is to be accomplished, creating elder-friendly communities has a logical and important role. Scharf et al. [7] suggest an important association between social connectedness and quality of life. They found that older people who rated their quality of life as “good” were less likely to experience social exclusion. For aging in place to happen successfully, with older adults being continually valued and integrated into community life, city officials, policy makers, and gerontological researchers will need to collaborate in order to move these ideas from research to reality. 
